# MAP-Kinase Activated Protein Kinase 2 Links Endothelial Activation and Monocyte/macrophage Recruitment in Arteriogenesis

**DOI:** 10.1371/journal.pone.0138542

**Published:** 2015-10-02

**Authors:** Anne Limbourg, Johann von Felden, Kumaravelu Jagavelu, Kashyap Krishnasamy, L. Christian Napp, Piyushkumar R. Kapopara, Matthias Gaestel, Bernhard Schieffer, Johann Bauersachs, Florian P. Limbourg, Udo Bavendiek

**Affiliations:** 1 Vascular Medicine Research, Dept. of Nephrology and Hypertension, Hannover Medical School, Hannover, Germany; 2 Integriertes Forschungs- und Behandlungszentrum Transplantation (IFB-Tx), Hannover Medical School, Hannover, Germany; 3 Dept. of Cardiology & Angiology, Hannover Medical School, Hannover, Germany; 4 Dept. of Biochemistry, Hannover Medical School, Hannover, Germany; University of Amsterdam Academic Medical Center, NETHERLANDS

## Abstract

Arteriogenesis, the growth of natural bypass arteries, is triggered by hemodynamic forces within vessels and requires a balanced inflammatory response, involving induction of the chemokine MCP-1 and recruitment of leukocytes. However, little is known how these processes are coordinated. The MAP-kinase-activated-proteinkinase-2 (MK2) is a critical regulator of inflammatory processes and might represent an important link between cytokine production and cell recruitment during postnatal arteriogenesis. Therefore, the present study investigated the functional role of MK2 during postnatal arteriogenesis. In a mouse model of hindlimb ischemia (HLI) MK2-deficiency (MK2KO) significantly impaired ischemic blood flow recovery and growth of collateral arteries as well as perivascular recruitment of mononuclear cells and macrophages. This was accompanied by induction of endothelial MCP-1 expression in wildtype (WT) but not in MK2KO collateral arteries. Following HLI, MK2 activation rapidly occured in the endothelium of growing WT arteries *in vivo*. *In vitro*, inflammatory cytokines and cyclic stretch activated MK2 in endothelial cells, which was required for stretch- and cytokine-induced release of MCP-1. In addition, a monocyte cell autonomous function of MK2 was uncovered potentially regulating MCP-1-dependent monocyte recruitment to vessels: MCP-1 stimulation of WT monocytes induced MK2 activation and monocyte migration *in vitro*. The latter was reduced in MK2KO monocytes, while *in vivo* MK2 was activated in monocytes recruited to collateral arteries. In conclusion, MK2 regulates postnatal arteriogenesis by controlling vascular recruitment of monocytes/macrophages in a dual manner: regulation of endothelial MCP-1 expression in response to hemodynamic and inflammatory forces as well as MCP-1 dependent monocyte migration.

## Introduction

The growth and maturation of small collateral arteries (CA) into conduit vessels, termed arteriogenesis, is critical for restoration of blood flow to ischemic organs. This process is triggered by intravascular hemodynamic forces, involves the production of inflammatory cytokines and chemokines, and requires monocyte and macrophage recruitment to the growing vessel wall [1,2,3,4]. The chemokine MCP-1, the cognate ligand for CCR2, exerts profound effects on monocyte and macrophage migration and is critical for arteriogenesis [5]. MCP-1 release is induced in endothelial cells by hemodynamic forces, such as cyclic stretch [6,7,8], and is required for recruitment of monocytes and macrophages to growing collaterals. However, intracellular signaling modules regulating this coordinated response from hemodynamic signals to leukocyte migration and recruitment to the vessel wall are largely unknown.

The mitogen-activated protein kinase (MAPK)-activated protein kinase 2 (MK2) represents an important intracellular signaling protein mediating inflammatory processes. Recent studies demonstrate a central role of MK2 in the production of proinflammatory mediators (e. g. TNFα, IL-6) as well as for cell migration by modulating actin remodelling and stress fiber formation [9]. The important functional role of MK2 for inflammatory processes was most clearly revealed in MK2-deficient mice employing different models of inflammatory diseases *in vivo* [9]. These studies also demonstrated an essential role of MK2 for cytokine-induced expression of MCP-1 in endothelial cells [10,11]. As balanced inflammatory processes such as expression of MCP-1 as well as migration of monocytes are both required for postnatal arteriogenesis, the present study investigated the functional role of MK2 in postnatal arteriogenesis employing a murine model of hind limb ischemia (HLI).

## Material and Methods

### Hind limb ischemia model

This study was conducted conforming to the German Law for the Protection of Animals and the NIH Guide for the Care and Use of Laboratory Animals. It was approved by the Institutional Animal Care and Use Committee (IACUC) of Hannover Medical School and authorities of the government of lower-saxony (Niedersächsisches Landesamt für Verbraucherschutz und Lebensmittelsicherheit, Approval #13/1194). Every effort was made to minimize stress and discomfort of the animals during the experiments. Animals were housed in an air-conditioned animal facility in separately ventilated cages and fed *ad libitum* with balanced food and water. They were maintained on a 12 h light/darkness cycle. Health status was regularly determined at baseline without any detection of abnormalities/diseases before the experiments. Induction of hind limb ischemia and blood flow analysis by laser-doppler perfusion imaging were performed in wildtype (WT) and MK2-deficient mice [12] (global knock-out, back-crossed at least 10 generations to the genetic background B57BL/6J) as described previously [13,14]. Briefly, mice were anesthetized by intraperitoneal injection of a mixture of ketamine (2 mg/kg body weight) and xylazine (13 mg/kg body weight) and the femoral artery was ligated distal to the origin of the deep femoral artery and proximal to the popliteal artery. Blood flow measurements in mouse feet were performed on 37°C heated pads before and immediately after surgery, and on post-operative days 3, 7, 14, and 21 using a laser Doppler perfusion imager (PIM II, Perimed, Sweden). Perfusion was expressed as the ratio of ligated-to-nonligated side. No important adverse events before final analysis were detected demanding early termination of the experiment.

### Tissue sampling and analysis

Collateral arteries from the deep femoral artery were analyzed in cryo-sections of OCT-embedded paraformaldehyde fixed semimembranous muscles by histomorphometry and laser scanning confocal microscopy as described [13]. Histomorphometry of collateral arteries was performed on at least 5 H&E stained sections of semimembranous muscles using Axiovision Rel 4.4 software and an Axiovert 200 microscope (Carl Zeiss Microscopy). The arterial wall area was calculated by subtracting the lumen area from the outer circumference of the tunica media. Laser scanning confocal microscopy after immunostaining was performed with a TCS SP2 AOBS (Leica Microsystems) employing specific antibodies against phospho-MK2 (Cell Signaling Technologies), CD31 (BD Biosciences), F4/80 (Serotec), α-smooth muscle cell actin (Sigma) and MCP-1 (Santa Cruz Biotechnologies) as well as DAPI for nuclear staining.

### Quantitative RT-PCR

RNA from fresh murine muscle tissues (100 mg) was isolated after mechanical disruption with the Nucleospin RNA II Kit (Macherey Nagel GmbH) as per manufacturer’s protocol. After purity and quality check, RNA was reverse transcribed into cDNA with Invitrogen RT Kit (Invitrogen GmbH, Germany) according to manufacturer’s instructions. For gene expression analysis, real-time primers were designed using the software Primerquest (IDT) and PrimerBLAST (NCBI) following general guidelines for primer design and synthesized by Eurofins GmbH. Quantitative real-time PCR was performed using *Ccl2*-specific primers (Forward: TGGGGCGTTAACTGCATCTGG, Reverse: TGGGCCTGCTGTTCACAGTT, Final concentration: 10 pmol/μL) and FastStart Essential DNA Green Master mix (Roche) in special optical 96-well plates using a spectrofluorometric thermal cycler (Lightcycler, Roche) according to manufacturer’s instructions. Expression of each specific gene was normalized to expression of *Rps9* and fold change in expression was calculated by the CT method [15].

### Isolation and culture of primary murine endothelial cells

Isolation and culture of primary murine endothelial cells from WT and MK2-deficient mice (age 8–10 weeks) were performed as described previously [16]. Briefly, endothelial cells were isolated from mouse lungs by magnetic immunosorting employing specific antibodies against CD31 and ICAM-2 (Dynal/Invitrogen). Cells were seeded in 6 well plates pre-coated with fibronectin in mEC growth medium [DMEM/F -12 (Gibco) + supplement ECGS/H, (PromoCell) + 10% FCS + 1% penicillin/streptomycin] and incubated at 37°C in 5% CO_2_. For experiments endothelial cells were plated on six-well plates coated with gelatine. Before exposure to mechanical stretch or cytokines (IL-1β, TNFα; Pierce) endothelial cells were starved in endothelial basal medium (DMEM/F-12, Gibco) supplemented with 0.5% FCS for 4 h.

### Mechanical stretch

Mechanical stretch of cells was performed as described previously [17]. In brief, endothelial cells were plated on six-well silicone elastomer plates (Bioflex, Flexcell International Corp.) coated with gelatine. Confluent endothelial cell monolayers were exposed to continuous cycles of stretch and relaxation (0.5 Hz) by use of the Flexercell Strain Unit FX-3000 (Flexcell International Corp.) for indicated times; a maximum of 10% radial stretch of the membrane was applied.

### Immunoblotting & ELISA

Immunoblotting was performed after separation of proteins by standard SDS-PAGE as described [18] using specific antibodies against phopho-MK2 and MK2 (Cell Signaling Technologies). ELISA for MCP-1 concentration in cell culture supernatants was performed according to the manufacturer´s protocol (R&D Systems).

### Isolation of primary murine monocytes and transwell migration assay

Primary murine monocytes were isolated from peripheral blood after ficoll gradient density centrifugation followed by positive selection with CD11b-coupled magnetic microbeads (Miltenyi Biotech.) according to the manufacturer´s protocol. Migration of monocytes towards MCP-1 (10 ng/ml, R&D Systems) was investigated in a transwell migration assay employing dedicated cell culture chambers (8μm pore size, Corning, Costar) as described previously [19].

### Statistical analysis

Data are expressed as means ± SEM unless indicated otherwise. Groups were compared by unpaired student´s *t* tests or one-way ANOVA and ad hoc Tukey-Kramer multiple comparisons using InStat software (Graphpad Software). A p-value below 0.05 was considered statistically significant.

## Results

### MK2-deficiency impairs arteriogenesis and recruitment of macrophages to collateral arteries after hindlimb ischemia in mice

**Fig 1:**
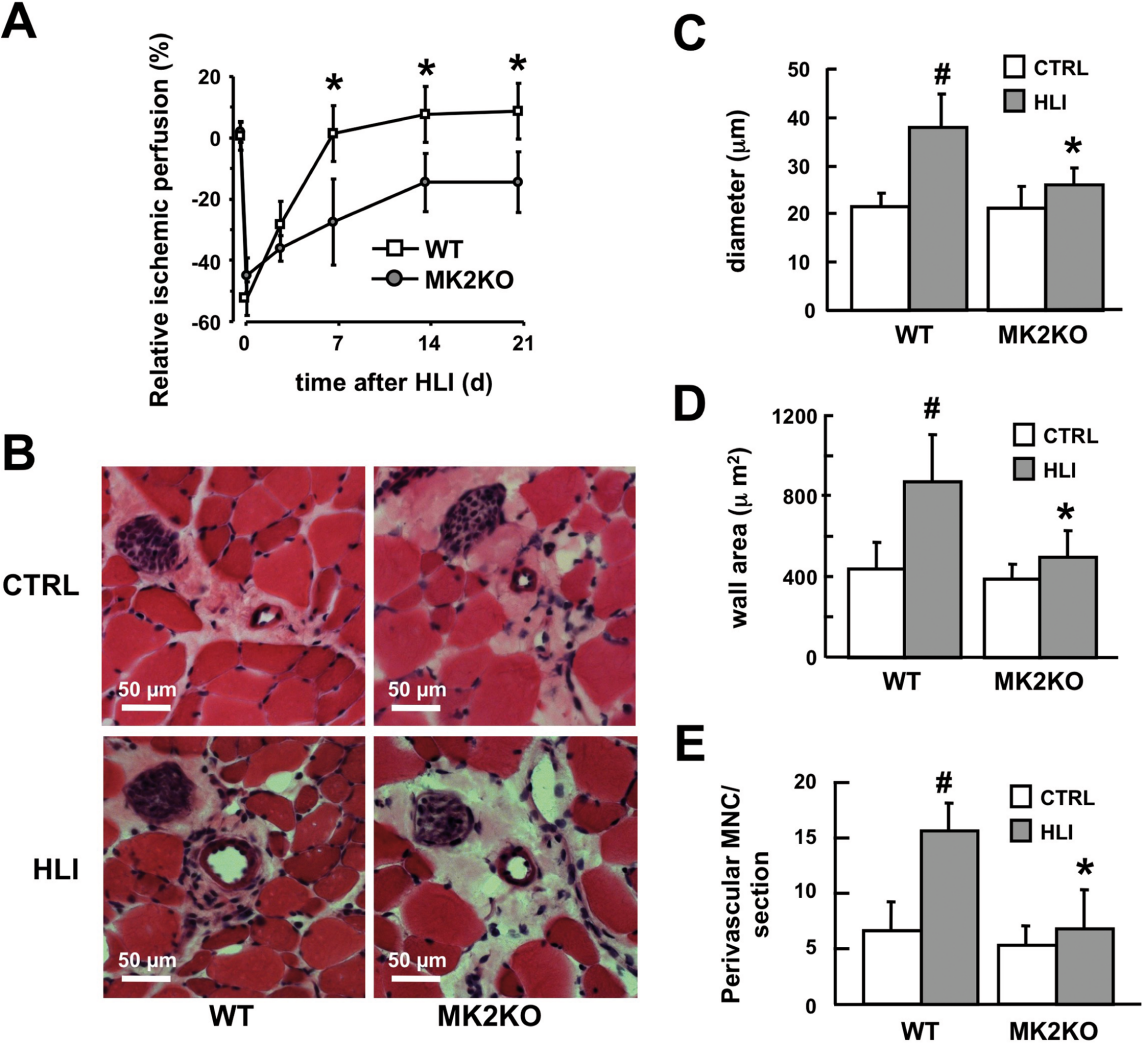
MK2 essentially regulates blood flow recovery after hind limb ischemia and growth of collateral arteries during postnatal arteriogenesis. A, blood flow was determined by laser-doppler-blood flow analysis in wildtype (WT) and MK2-deficient (MK2KO) mice after HLI at the indicated times (day 0 to 21 after arterial ligation). Perfusion is given as relative ischemic perfusion compared to blood flow before arterial ligation (*p< 0.01 WT vs. MK2KO, n = 11). B-E, after HLI (day 21 post arterial ligation) sections (B, H & E staining) of collateral arteries of the ischemic (HLI) and contralateral non-ischemic limb (CTRL) from WT and MK2KO were analyzed by histomorphometry for diameter (C), wall area (D) and number of perivascular mononuclear cells (E). (C-E), #*p*< 0.05 control vs. HLI, *p< 0.01 WT vs. MK2KO, n = 6).

**Fig 2:**
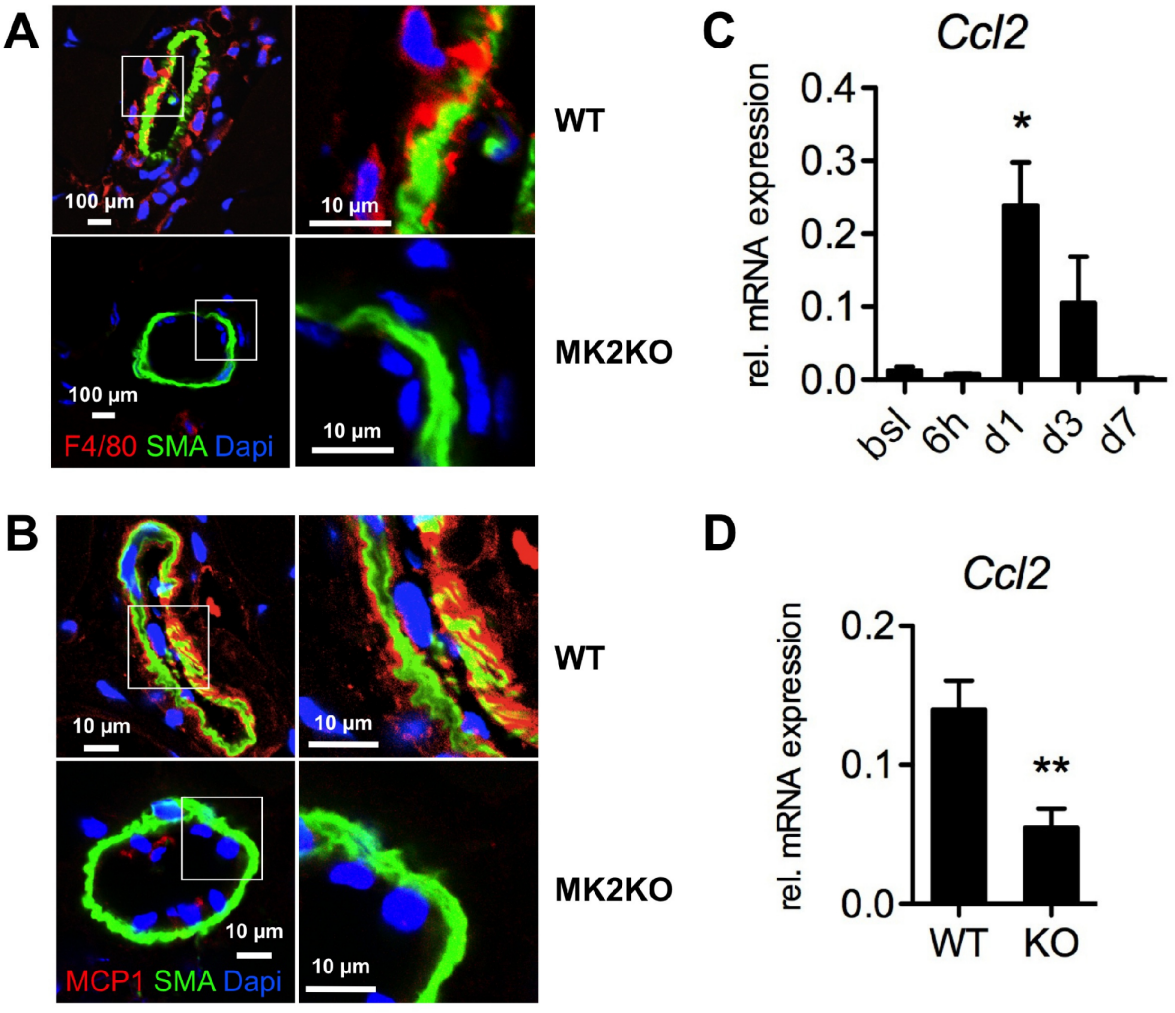
MK2 is essential for the vascular recruitment of macrophages and expression of MCP-1 during postnatal arteriogenesis. A-B, after hind limb ischemia sections of collateral arteries from the ischemic limb from WT and MK2KO were stained for macrophages (A) or MCP-1 (B) (red, F4/80 or MCP-1), α-smooth muscle cell actin (green, SMA) and cell nuclei (blue, DAPI) and analyzed by fluorescence laser scanning confocal microscopy. Representative staining of at least 3 different experiments (day 3 post arterial ligation) are shown. C, mRNA-expression of MCP-1 was determined in hind limb muscle from wildtype (WT) mice at baseline (bsl) and 6 h, day 1 (d1), days 3 (d3), and day 7 (d7) after HLI. Relative mRNA-expression determined by qRT-PCR is shown (*p< 0.05 baseline vs. day 1, n = 4–5). D, mRNA-expression of MCP-1 was determined in hind limb tissue from wildtype (WT) and MK2-deficient mice (MK2KO) at day 1 (d1) after HLI (*p< 0.01 WT vs. MK2KO, n = 6).

We first evaluated ischemic blood flow recovery and arteriogenesis in wildtype (WT) and homozygous MK2-deficient (MK2KO) mice in a mouse model of hindlimb ischemia (HLI) [14]. As determined by laser-doppler perfusion imaging, arterial ligation reduced distal hind limb blood flow to a comparable degree in WT and MK2KO mice. However, whereas blood flow to ischemic tissue rapidly and completely recovered in WT mice, perfusion recovery in MK2KO mice was slower and incomplete, never returning to baseline values (Fig 1A). Histomorphometric analysis in tissue sections revealed collateral remodeling in response to HLI in WT mice, demonstrated by an increase in collateral diameter and wall area, while MK2KO mice showed significantly impaired collateral remodeling (Fig 1B–1E). Furthermore, in WT mice HLI caused a pronounced perivascular accumulation of mononuclear cells around growing collaterals (Fig 1B–1E), which consisted mostly of F4/80 positive macrophages (Fig 2A, upper panels), which are known to be essential for proper arteriogenesis [20]. In contrast, mononuclear cell recruitment and accumulation of F4/80 positive macrophages to collateral arteries was severely impaired in MK2KO mice (Figs 1B, 1C & 2A, lower panels). To address whether MK2-dependent monocyte-macrophage accumulation is confined to the postischemic period we quantified the pre-ischemic content of perivascular tissue–resident mononuclear cells (MNC) and macrophages (F4/80^+^ cells), which demonstrated comparable cell counts in collateral arteries from non-ischemic limbs of WT and MK2KO (S1 Fig).

Overall, these results indicate an important role for MK2 in postnatal arteriogenesis by regulating the growth of preformed collateral arteries as well as the perivascular recruitment of mononuclear cells, in particular macrophages during this process.

### MK2 is activated in the endothelium of collateral arteries during postnatal arteriogenesis and regulates MCP-1 expression

The chemokine MCP-1 is a major chemoattractant for monocytes and the MCP-1-CCR2 pathway is essential for postnatal arteriogenesis [5]. As the MK2KO phenotype resembled MCP-1 signaling-deficient phenotypes observed in models of arteriogenesis [5], we investigated whether MK2 activation might regulate vascular MCP-1 expression *in vivo*. In line with the marked perivascular accumulation of macrophages, WT collaterals revealed strong expression of endothelial MCP-1 after HLI (Fig 2B, upper panels). In contrast, expression of MCP-1 was virtually undetectable in MK2KO collateral arteries after HLI (Fig 2B, lower panels). To further investigate the relationship between MK2 and MCP-1 expression we analyzed *Ccl2* mRNA induction in hind limb muscles in response to HLI. In WT mice, significant elelvation of *Ccl2* mRNA-expression was observed one day after induction of HLI, which was significantly reduced in MK2KO mice (Fig 2C and 2D).

**Fig 3:**
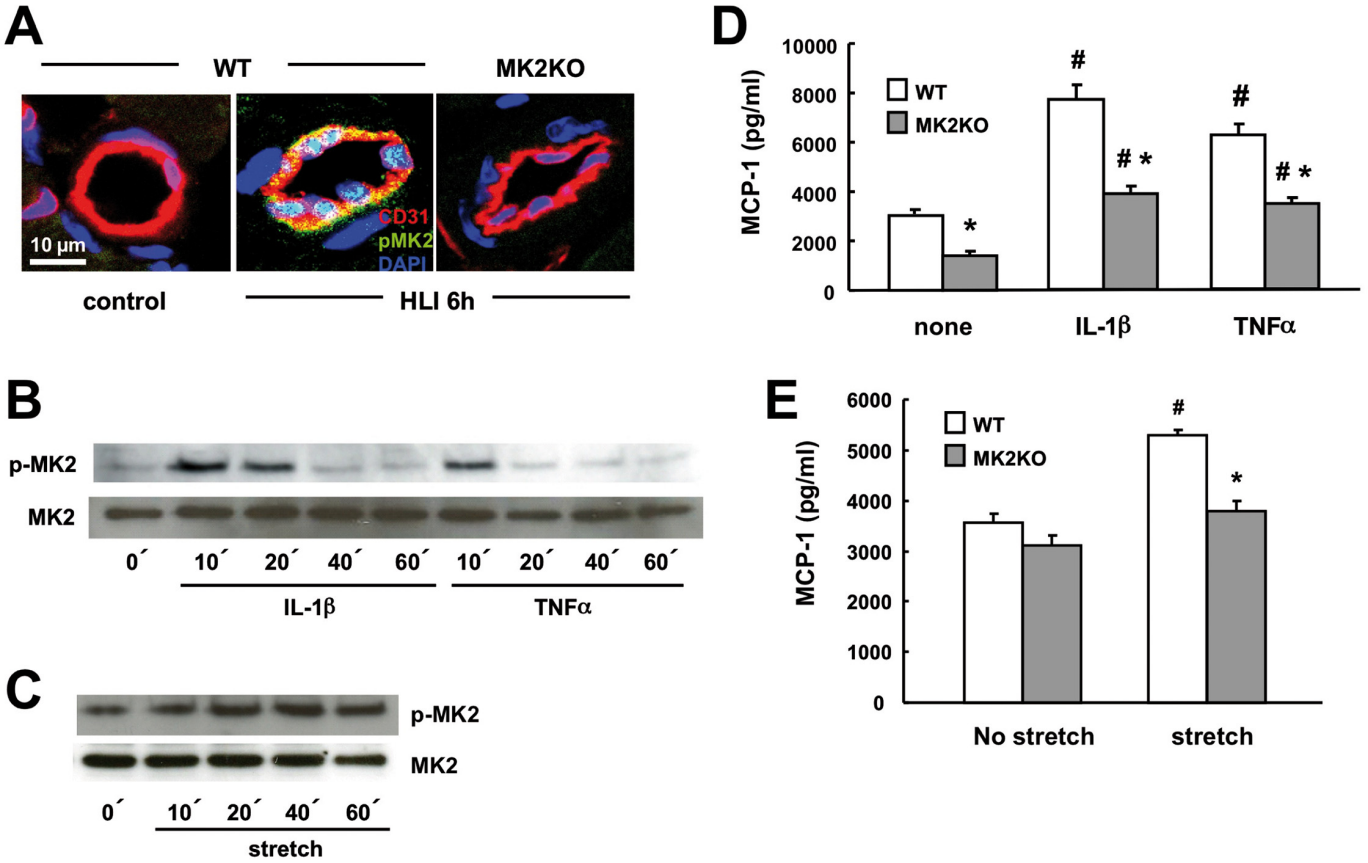
MK2 is activated in the endothelium of collateral arteries and mediates the expression of MCP-1 induced by pro-inflammatory cytokines as well as cyclic stretch in endothelial cells. A, after hind limb ischemia (6 h after arterial ligation) sections of collateral arteries from the ischemic (HLI) and the contralateral non-ischemic limb (control) of wildtype mice (WT) and MK2-deficient mice (MK2KO) were stained for activated phospho-MK2 and analyzed by fluorescence laser scanning confocal microscopy (phopho-MK2, green), endothelial cells (red, CD31), nuclear staining (DAPI, blue). Representative stainings of at least 3 different experiments are shown. B-E, cultured primary murine endothelial cells isolated from WT and MK2KO mice were stimulated with IL-1β (10 ng/ml), TNF-α (50 ng/ml) or cyclic stretch (0.5 Hz, 10% elongation) for 0–60 min. (B-C) or 6 h (D-E). Activation of MK2 was determined by immunoblotting for phospho-MK2 (B-C) or release of MCP-1 protein was quantified by ELISA in cell culture supernatants (D-E). Blots of experiments employing endothelial cell isolations from 5 different mice of each genotype are shown (B-C). #*p*< 0.05 control vs. cytokine or cyclic stretch, *p< 0.05 WT vs. MK2KO, n = 4–6).

To elucidate the cellular source of MK2-dependent MCP-1 expression required for arteriogenesis we analyzed MK2 activation in response to HLI *in situ* by anti-phospho-MK2 staining and confocal laser scanning microscopy. MK2 activation was not detectable in collateral arteries of WT mice under steady state conditions. In contrast, MK2 activation occurred in collateral arteries of WT mice already within 6 h following HLI, which was confined to the endothelium and not detectable in the smooth muscle cell rich arterial media. MK2 activation was not detectable in homozygous MK2KO mice in response to HLI, demonstrating the specificity of the analysis (Fig 3A).

Endothelial cells (EC) are a major source of MCP-1 during vascular inflammation. Herein, MCP-1 expression is induced by pro-inflammatory cytokines as well as by hemodynamic forces, such as cyclic stretch [6,7,8]. Predominant activation of MK2 in the endothelium but not in smooth muscle cells (SMC), which we observed in collateral arteries after HLI (Fig 3A), suggested an important differential function of MK2 for MCP-1 expression during arteriogenesis. Therefore, we tested whether MK2 activation is required for MCP-1 induction in EC by subjecting primary mouse EC isolated from WT and MK2KO mice to cyclic stretch and pro-inflammatory cytokines *in vitro*. IL-1β and TNFα, but also cyclic stretch activated MK2 in a time-dependent manner (Fig 3B & 3C) and induced the release of MCP-1 from EC (Fig 3D & 3E). Importantly, MK2-deficiency significantly reduced the MCP-1 release induced by cytokines and cyclic stretch in EC (Fig 3D & 3E).

### MK2 is activated in monocytes and mediates MCP-1 induced monocyte migration

**Fig 4:**
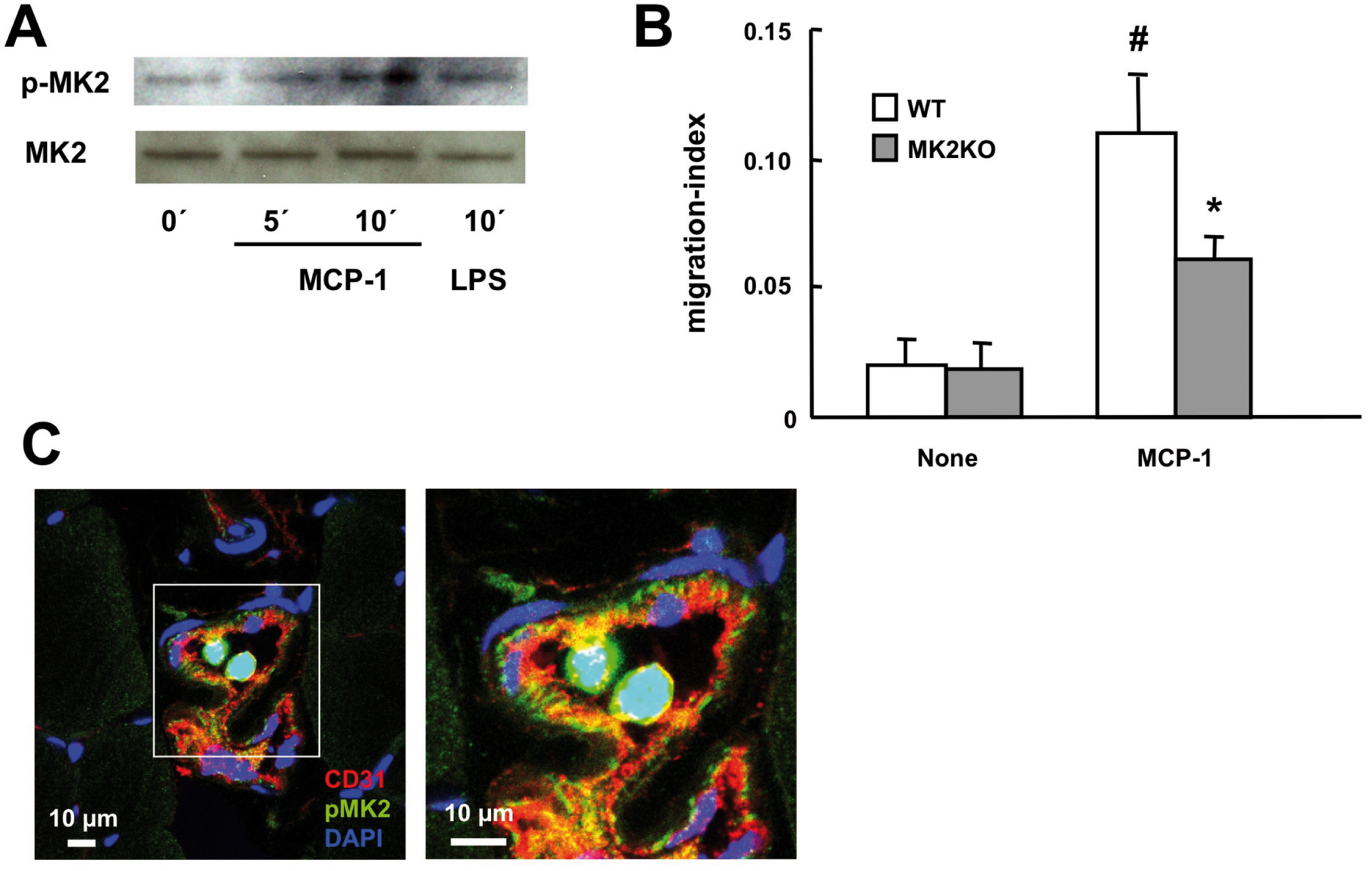
MK2 is activated in monocytes recruited to collateral arteries and mediates MCP-1 induced monocyte migration. A, Primary murine monocytes isolated from WT mice were stimulated with MCP-1 (10 ng/ml) or LPS (100ng/ml) for the indicated times and activation of MK2 was determined by immunoblotting for phospho-MK2. Representative blots of two independent experiments each employing monocyte isolations from 3 different mice are shown. B, Primary murine monocytes isolated from WT and MK2KO were given into the upper chamber of a transwell cell culture dish and number of cells migrated towards MCP-1 (10 ng/ml) in the lower chamber was quantified after 24 h and normalized to the total number of cells given into the upper chamber (migration-index). #*p*< 0.05 control vs. MCP-1, *p< 0.05 WT vs. MK2KO, n = 5). C, after hind limb ischemia (6 h after arterial ligation) sections of collateral arteries from the ischemic limb of WT mice were stained for activated phospho-MK2 and analyzed by fluorescence laser scanning confocal microscopy (phospho-MK2, green; endothelial cells, red, CD31; nuclear staining, blue, DAPI). Representative stainings of at least 3 different experiments are shown.

As MK2 is also involved in cell migration [9], an additional role of MK2 in the regulation of MCP-1 induced monocyte migration during arteriogenesis was also possible. *In vitro*, MCP-1 stimulation activated MK2 in isolated mouse monocytes (Fig 4A). While MCP-1 induced migration of WT monocytes in a transwell migration assay, this effect was significantly reduced in MK2KO monocytes (Fig 4B). Furthermore, immunostaining revealed MK2 activation in monocytes recruited to the endothelium in growing collateral arteries *in vivo*, corroborating its intrinsic role in monocyte recruitment during arteriogenesis (Fig 4C).

## Discussion

Postnatal arteriogenesis, a critical process for ischemic recovery, requires the integration and coordination of hemodynamic vascular signals as well as a balanced inflammation involving chemokine production and leukocyte recruitment for functional maturation and growth of collateral arteries. Combining *in vivo* and *in vitro* studies, our study—for our knowledge for the first time—provides genetic evidence that a stress-kinase, MAP kinase activated protein kinase 2 (MK2), is a critical regulator of arteriogenesis. The early and selective activation of MK2 observed after induction of HLI in the endothelium of collateral arteries clearly proceeded MCP-1 expression, which started to increase significantly at day 1 after HLI but not at 6 h, when activation of MK2 was detected. This points to a crucial role of MK2 in the connection of hemodynamic forces to the production of MCP-1, a well known critical regulator of arteriogenesis [5,21]. This is also supported by the remarkably similar phenotypes of mice deficient for the cognate receptor of MCP-1, CCR-2 [5], and MK2 in the mouse model of HLI. Both show strongly impaired growth of collaterals and pericollateral recruitment of macrophages associated with marked impairment of ischemic blood flow recovery. Inflammatory cytokines as well as hemodynamic forces, such as cyclic stretch, triggered MK2 activation in EC *in vitro*. Consistently, MK2 activation occurred in the endothelium during the immediate-early phase after HLI, which is dominated by high hemodynamic stress in collateral arteries [2]. The observed activation of MK2 in endothelial cells *in vitro* and *in vivo* was accompanied by a stretch-induced expression of MCP-1 in endothelial cells, which is in accordance with previous studies [6,7,8]. Induction of MCP-1 expression by hemodynamic forces in vascular smooth muscle cells but not endothelial cells was described to be a critical determinant for the initiation of arteriogenesis [7]. However, this conclusion was entirely based on data obtained in perfusion models *in situ* mimicking the hemodynamic changes in collateral arteries by employing second order branches of the mesenteric artery and mechanical denudation of the endothelium [7]. In this model, expression of MCP-1 was only localized to the media but not to the endothelium, which is conceivable since the endothelial layer was denuded artificially before. Moreover, results from this study cannot be transferred to the model of HLI due to profound differences in the nature of the models. Accordingly and in line with our results, a marked increase of MCP-1 localized to collateral arteries was also demonstrated in the model of HLI previously [8]. As activation of MK2 was clearly localized to the endothelium but not to the media of collateral arteries in our model of HLI, MK2-dependent release of MCP-1 in arteriogenesis is most likely specifically mediated by endothelial but not smooth muscle cells. In addition to hemodynamic forces, inflammatory cytokines (e. g. IL-1β, TNFα), which are released by differentiated and activated monocytes/macrophages after recruitment to collateral arteries, might enhance and perpetuate the vascular recruitment of inflammatory cells, namely monocytes/macrophages, via activation of MK2 and induction of MCP-1 in the endothelium. Besides its crucial role for MCP-1 expression in the vascular wall of growing collateral arteries, MK2 also seems to be important for MCP-1-dependent monocyte migration itself. This was demonstrated by MCP-1-dependent activation of MK2 in monocytes and impaired MCP-1 induced migration in MK2-deficient monocytes *in vitro*. Our data are supported by previous studies investigating the functional role of the direct upstream activator of MK2, p38 MAPK, for MCP-1 induced chemotaxis and transendothelial migration of monocytes. These studies demonstrated MCP-1 dependent activation of p38 MAPK in monocytes as well as partial inhibition of MCP-1 induced monocyte chemotaxis and transendothelial migration by pharmacological inhibition of p38 MAPK [22,23]. This is in line with the inhibition of MCP-1 induced monocyte migration by MK2-deficiency, which we observed in our experiments. The impact of MK2-deficiency on MCP-1 dependent monocyte recruitment and migration is most likely based on the well characterized function of MK2 as a central regulator of cell migration via a MK2-dependent regulation of heat shock protein 25/27 targeting actin remodelling and stress fiber formation [9].

**Fig 5:**
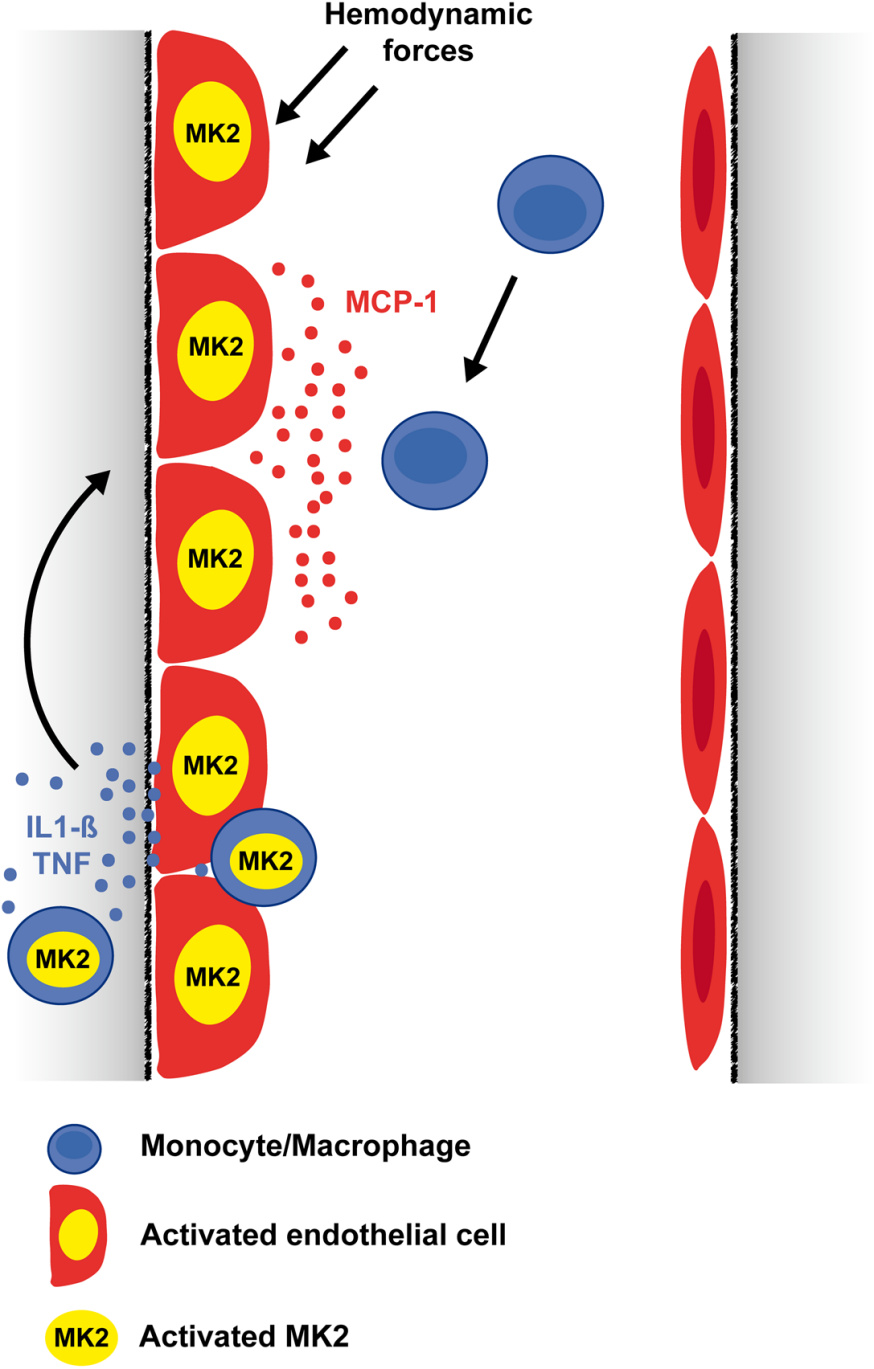
Model of MK2-regulated postnatal arteriogenesis. MK2 controls vascular recruitment of monocytes/macrophages during postnatal arteriogenesis in a dual manner: regulation of endothelial MCP-1 expression in response to hemodynamic and inflammatory forces as well as MCP-1-dependent monocyte migration.

Based on our data *in vitro* and *in vivo* we propose the following model of MK2 action at different levels of the arteriogenic process resulting in its crucial function in arteriogenesis (Fig 5). First, MK2 activation by hemodynamic forces mediates endothelial expression of MCP-1. In the next step, MCP-1 released from activated endothelial cells mediates activation of MK2 in monocytes resulting in their vascular recruitment and migration. Finally, monocytes recruited to collateral arteries release a variety of inflammatory mediators such as cytokines (e. g. IL-1β, TNFα). These cytokines very potently induce MCP-1 expression in endothelial cells in a MK2-dependent fashion, in turn causing further vascular recruitment of monocytes/macrophages. Thereby, the arteriogenic process is perpetuated until hemodynamic forces as the initiating trigger of arteriogenesis fade as the vessel grows.

### Conclusions

In conclusion, MK2 seems to represent a central regulator of postnatal arteriogenesis by controlling vascular recruitment of monocytes/macrophages in a dual manner: regulation of endothelial expression of MCP-1 in response to hemodynamic and inflammatory forces as well as MCP-1-induced migration of monocytes. Further studies investigating upstream and downstream targets of MK2 will help to elucidate its spatial and temporal role during postnatal arteriogenesis in more detail.

## Supporting Information

S1 Checklist The ARRIVE Guidelines Checklist—Animal Research: Reporting in Vivo Experiments. (PDF)

S1 Fig Comparable pre-ischemic content of perivascular tissue–resident mononuclear cells (MNC) and macrophages (F4/80^+^ cells) in wildtype and MK2-deficient collateral arteries. (TIF)
