# Supplementary figures and images for: MAP-Kinase Activated Protein Kinase 2 Links Endothelial Activation and Monocyte/macrophage Recruitment in Arteriogenesis
Source: PLoS One. 2015 Oct 2;10(10):e0138542. doi: 10.1371/journal.pone.0138542 (PMC4592267; doi:10.1371/journal.pone.0138542)

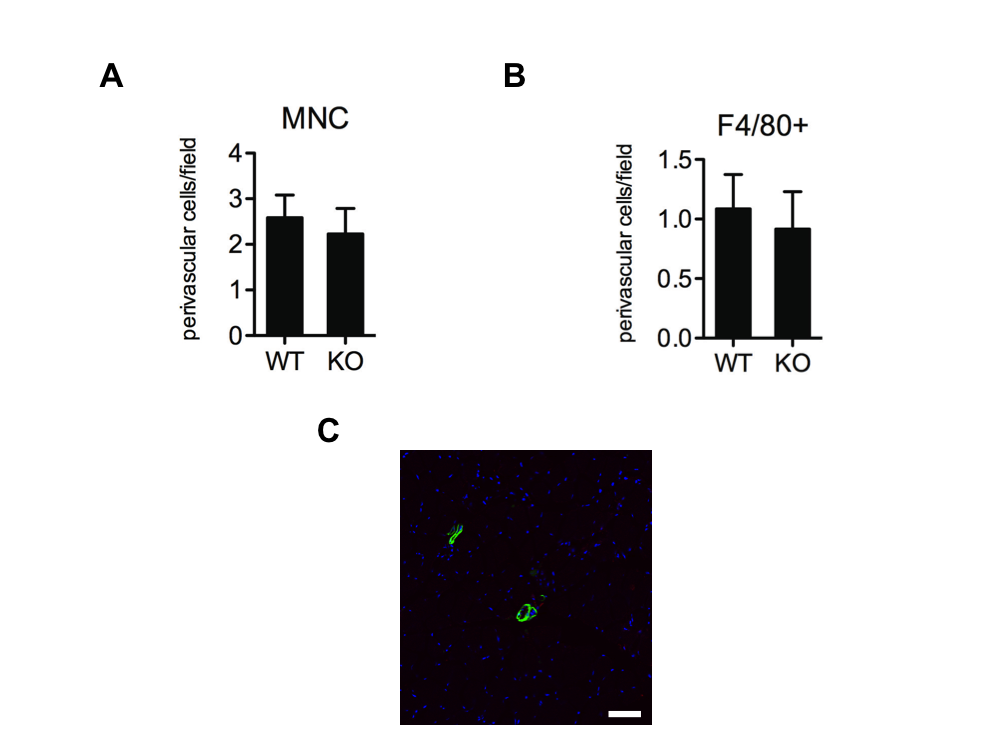

Supplement: S1 Fig — (TIF) [file pone.0138542.s002.tif]
